# Global regulatory architecture of human, mouse and rat tissue transcriptomes

**DOI:** 10.1186/1471-2164-14-716

**Published:** 2013-10-20

**Authors:** Ajay Prasad, Suchitra Suresh Kumar, Christophe Dessimoz, Stefan Bleuler, Oliver Laule, Tomas Hruz, Wilhelm Gruissem, Philip Zimmermann

**Affiliations:** 1 Department of Biology, ETH Zurich, 8092 Zurich, Switzerland; 2 Nebion AG, Hohlstrasse 515, 8048 Zurich, Switzerland; 3 Swiss Institute of Bioinformatics, Universitätstr. 6, 8092 Zurich, Switzerland; 4 Department of Computer Science, ETH Zurich, 8092 Zurich, Switzerland; 5 University College London, Gower Street, London, WC1E 6BT, UK

## Abstract

**Background:**

Predicting molecular responses in human by extrapolating results from model organisms requires a precise understanding of the architecture and regulation of biological mechanisms across species.

**Results:**

Here, we present a large-scale comparative analysis of organ and tissue transcriptomes involving the three mammalian species human, mouse and rat. To this end, we created a unique, highly standardized compendium of tissue expression. Representative tissue specific datasets were aggregated from more than 33,900 Affymetrix expression microarrays. For each organism, we created two expression datasets covering over 55 distinct tissue types with curated data from two independent microarray platforms. Principal component analysis (PCA) revealed that the tissue-specific architecture of transcriptomes is highly conserved between human, mouse and rat. Moreover, tissues with related biological function clustered tightly together, even if the underlying data originated from different labs and experimental settings. Overall, the expression variance caused by tissue type was approximately 10 times higher than the variance caused by perturbations or diseases, except for a subset of cancers and chemicals. Pairs of gene orthologs exhibited higher expression correlation between mouse and rat than with human. Finally, we show evidence that tissue expression profiles, if combined with sequence similarity, can improve the correct assignment of functionally related homologs across species.

**Conclusion:**

The results demonstrate that tissue-specific regulation is the main determinant of transcriptome composition and is highly conserved across mammalian species.

## Background

Mouse and rat are frequent mammalian models in biomedical research to learn more about a disease, its diagnosis and its treatment. Comparing results obtained from mammalian models with those from human samples is essential to estimate conservation of molecular mechanisms across species and to refine prediction models. Cross-species comparisons can be carried out at several levels such as genomic or protein sequences, molecular abundances, or phenotypes. On the sequence level, the conservation of protein sequence between species has been extensively studied. A more recent development has been the study of genomic alterations, some of which appear to be highly relevant in translational efforts from model organisms to human [1]. For example, several genetic mutations in acute promyelocytic leukemia have been shown to be conserved between human and mouse and are expected to be relevant for the disease [2]. On the level of molecular abundance, protein or transcript expression are usually assessed either by comparing absolute abundances between samples (e.g. time-course or tissue type comparisons) or abundance ratios (e.g. response to a perturbation or disease, given as a ratio of an experimental versus a control condition). Several studies exist comparing transcript abundance in various tissues. Most of these studies were restricted to comparing the human and mouse transcriptomes, thereby limiting the interpretation to a bilateral relationship without evidence from further organisms [3-7]. Comparing exclusively human and mouse lacks a measure for estimating inter-species distance in transcriptome composition. Including rat as a close relative to mouse is crucial to assess the quality of the differences measured between mouse and human.

In contrast to tissue expression studies, the comparison of responses to perturbations has been much more difficult because available data consists of experiments carried out under conditions that are not easily comparable. In fact, from all public data currently available from gene expression repositories, very few experiments can be considered equivalent between human and mouse, such as cell cultures from the same tissue of origin and treated with the same chemical at identical concentration ranges and in a similar experimental setup. Therefore, comparisons have remained primarily descriptive, for example on the level of correlation network structures between species [8], assuming that the composition of data has little effect on overall network structure.

Recent comparative genomics efforts have led to a better understanding of conservation of gene expression between human and rodents. Nevertheless, there is still much debate about which regulatory aspects are conserved between gene homologs of different species. Earlier studies comparing the expression patterns of orthologous gene pairs in different tissues showed conflicting results. For example, some studies suggested that orthologous genes have dissimilar expression patterns [3,4,9-11], while others reported congruent expression profiles [5-7,12-17]. Reasons for this discrepancy could be local experimental effects due to low coverage of genes or conditions, technological and methodological choices, probe quality, data normalizaton issues, or different methods to identify orthologs between species.

In this work, we overcome these limitations by combining three mammalian species, a large set of tissue types, two independent datasets per organism, and a data preparation methodology that delivers highly representative and robust expression values from a very large set of original data. Specifically, we integrated high quality human, mouse and rat data from more than 33,900 Affymetrix expression arrays across six microarray platforms (3 species × 2 platforms per species) and across at least 55 overlapping tissue types between each set of platforms. The original data for this study was obtained from Genevestigator [18], a database of manually annotated, quality controlled and globally normalized public gene expression experiments.

The study was carried out on two distinct microarray platforms per species in order to assess the validity of the results across platforms and between independent experimental compositions. We grouped the platforms into two platform sets, SET 1 including arrays with lower transcript coverage (Affymetrix HG-U133A (20k), MG-U74Av2 (12k), and RG-U34 (8k)) and SET 2 including more recent full genome arrays (Affymetrix Human133 Plus 2.0 (47k), Mouse430 2.0 (40k) and Rat230 2.0 (31k)). To further minimize biases that can occur in our comparison between species, we carried out the following: 

• Overlap of tissue types: for all three species, we created data matrices with identical composition of tissues within each set.

• Single vector per tissue type: for each tissue type and microarray platform, we calculated a single representative expression vector based on all samples annotated with this tissue type.

• Selection of orthologs: we used OMA [19], a state-of-the-art orthology prediction algorithm, to obtain gene ortholog clusters between human, mouse and rat.

• Selection of probe sets: we excluded, wherever possible, probe sets targeting multiple transcripts, keeping only highly specific probe sets.

• Data normalization: a global normalization was performed across all data from a given microarray platform (see Methods section). Additionally, each probe set was normalized across all tissue types to yield a standardized representation of tissue specificity.

This resulted in highly robust datasets representing tissue-specific expression for human, mouse and rat. These highly standardized and quality tested datasets allowed us to conveniently address the following hypotheses: 

1. Hypothesis 1: The global architecture of tissue expression is conserved between human and rodent species.

2. Hypothesis 2: Orthologs of more closely related species have a higher tissue expression correlation.

3. Hypothesis 3: Tissue expression profiling can improve the mapping of functional orthologs.

## Results and discussion

### Architecture of tissue expression

**Figure 1:**
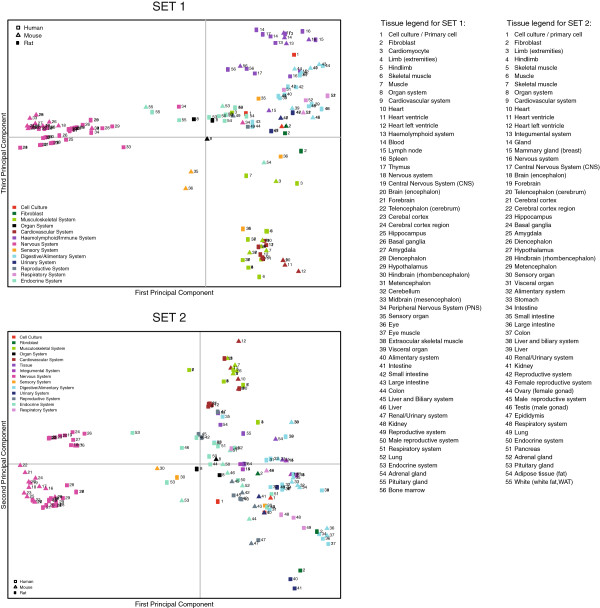
**Principle Component Analysis (PCA) of human, mouse and rat tissue expression profiles based on two generations of Affymetrix expression arrays shown as SET 1 (upper plot: Affymetrix HG-U133A, MG-U74Av2, and RG-U34 arrays) and SET 2 (lower plot: Affymetrix Human133 Plus 2.0, Mouse430 2.0, and Rat230 2.0 arrays).** A common set of 56 and 55 tissue types, respectively, is represented for each organism. Each tissue type is a single mean expression vector processed from all samples annotated as such in the Genevestigator database. Species are represented by symbols and tissue types are numbered. Tissue types were grouped according to organ systems that are represented by different colors. Related tissues clustered together into an overall consistent architecture between the three species. The two generations of microarrays yielded very similar results, despite being composed of independent and differerent sets of published experiments.

In order to evaluate the global architecture of tissue expression between human, mouse and rat gene orthologs (hypothesis 1), we performed a Principal Component Analysis (PCA) on SET 1 and SET 2, each containing 2127 and 8954 orthologous gene clusters represented by 56 and 55 tissue types, respectively (Figure 1). The original data is available in Additional file 1 (SET1) and Additional file 2 (SET 2).

We hypothesized that biologically related tissues would cluster together, while unrelated tissues would be located more distantly on the plot. This is assumed to be true if for each tissue type, a representative vector of expression for that tissue can be generated; here, we calculated an average vector from all samples annotated with a given tissue type, irrespective of the nature of the experiment. The PCA projections revealed that biologically related tissues indeed clustered close to each other. Furthermore, the clustering was highly similar between the two platform sets of a given organism, despite completely different experiment compositions. The global architecture was strikingly similar between the three species studied, both in SET 1 and SET 2. In contrast to a previous study [6], in which mouse had a similar overall structure as human but was scaled differently, our results revealed an almost identical architecture for human, mouse and rat, both in the scale and in the clustering of the projected individual tissue types. Considering human data alone, the results were similar to those obtained by other studies (e.g. [6,20], although here each category in the plot represents an average vector aggregated from a population of samples rather than plotting individual samples in the PCA.

The first principal component separated distinctly all central nervous system (CNS) tissues from all other body parts (limb, muscles, cell cultures and all inner organs). This suggests that all tissues of neural origin possess a major subset of genes that are strongly differentially expressed relative to tissues of other origin. For each species, the other principal components segregated all other tissue types (i.e. not of neuronal origin) into clusters of anatomical parts that have a common origin or physiology. For example, a variety of muscle tissues formed a distinct cluster which was located close to another cluster involving heart tissues (here labelled musculoskeletal and cardiovascular systems, respectively). On the opposite side of this component, related tissue clusters from the digestive, respiratory and renal systems were located. Interestingly, in SET 2, liver appears to be more closely located to the remaining tissues for mouse and rat in comparison to human. For SET 1, this is observed when principal component 1 is plotted against component 2. This result confirms previous findings in a comparison of human and mouse [6,21]. Finally, SET 1 also comprised tissues belonging to haemolymphoid or immune system, such as thymus, spleen, bone marrow, blood and lymph node. These tissues distinctly clustered together on the opposite side of the cardiovascular and musculoskeletal system (Figure 1A).

A particular case is the 'cell culture’ category. It appears distinct from the tissues from which these cells originated. The results suggest that bringing cells into culture causes a major shift in the transcriptome population. To assess the nature of this transformation more in detail, we identified a set of 177 genes exhibiting high expression in cell culture but minimal expression in all other normal tissues from SET 2. Gene Ontology enrichment analysis of these genes revealed that most of these genes belonged to apoptosis-related processes (see Additional file 3). The individual cell culture samples that were aggregated to obtain the average vector for the category 'cell culture’ contained primary cells from various organs, cell lines and also blood cells. We hypothesized that the apoptosis-related signature was mainly due to the presence of immune cells and immortalized cell lines. Therefore, to further refine our analysis and to compare uniquely primary cells to their organs of origin, we carried out an independent analysis using Genevestigator, in which we excluded cell lines and blood cells. This extensive search comparing 54 different cell culture types to over 200 normal tissue types revealed a set of 217 probe sets having strong expression in most cell culture categories, but minimal expression in all normal tissues (see Additional file 4). A Gene Ontology enrichment analysis of this set revealed that most of these genes were involved in biological processes related to extracellular structure organization, vasculature development, cell motility, biological adhesion and wound healing (see Additional file 5). Obviously, cells artificially extracted and isolated from their tissue context trigger the activation of processes to reestablish this context. These processes involve several hundred genes and therefore strongly influence the global transcriptome population of these cultures, as shown here and in previous work [20,22,23]. Interestingly, the activation of genes related to extracellular structure organization, vasculature development and cell motility was observed across a wide variety of cell types arising from functionally completely different organs. The shift between primary cells and their tissue of origin was in the same order of magnitude as between different organs and tissue types, raising questions about how cell culture can be used to model biological processes in vitro. Nevertheless, recent progress in our understanding of cell adhesion and interaction with neighboring cells are enabling cell culture models to better mimic in vivo processes [24,25].

**Figure 2:**
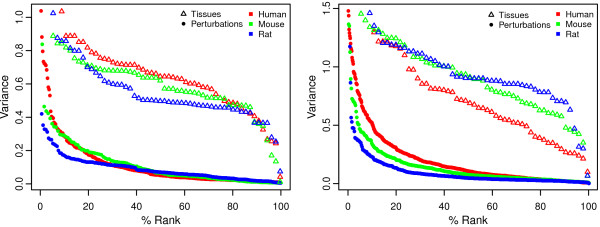
**Variance of expression logratios of 2127 genes (SET 1) and 8954 genes (SET 2) within different perturbations and tissue types.** For perturbations, logratios were calculated as experimental versus control samples, whereas for tissues they were calculated as the expression in a given tissue type relative to the average vector of all 56 or 55 tissue types, respectively. The variance of expression was sorted in decreasing order for SET 1 (left plot) and for SET 2 (right plot), for both perturbations and tissue types, and plotted against their percent ranking. Variances originating from tissue affiliation were signifcantly higher than variances arising from perturbations, except for a variety of cancers and drugs (see also Additional file 3).

In this study, we used representative expression vectors for each tissue that were summarized from all samples containing the corresponding tissue annotation, irrespective of the experimental conditions for each sample. Despite this diversity and unsystematic composition of experimental conditions, what is intriguing is the high degree of similarity of the tissue expression architecture between the three species and two sets of microarray platforms. Similarly, the tight clustering of tissue types having related biological functions is striking. This suggests that the variance due to experimental conditions may be significantly lower than the variance caused by tissue type. To evaluate this, we compared the log ratios of expression of tissue types relative to the mean of all tissues with log ratios of perturbations relative to the corresponding control samples. Figure 2(left plot) and 2(right plot) show that, generally, the variances originating from tissue affiliation are at least 10 times higher than the variances arising from perturbations, as measured from more than 500 different perturbation types (see Additional file 6). Exceptions to this rule were various cancers and several potent drugs and antibiotics. These results reveal that developmental processes and cell differentiation lead to end states with the activation/repression of a much larger number of genes and pathways than temporary responses to diseases or environmental cues. On this scale, cancer seems to result in intermediate, unstable states of transcriptomes.

### Conservation of expression regulation

**Figure 3:**
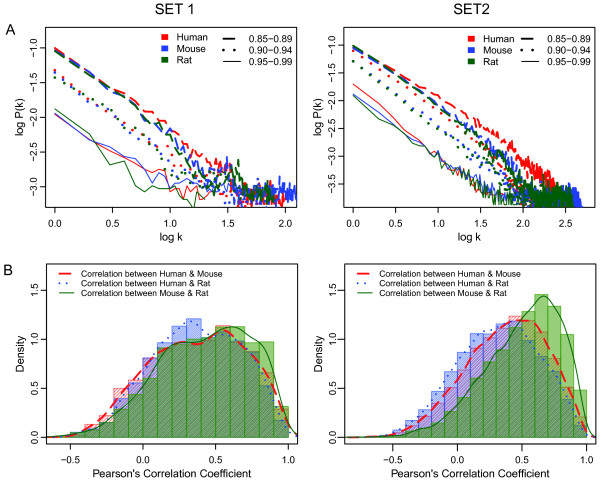
**A. Graph of degree distribution representing the fraction of nodes with k degrees (P(k)) versus degree (k) for SET 1 (left) and SET 2 (right).** For each microarray platform, the Pearson correlation network across the anatomical meta-profiles was calculated, and both k and P(k) were calculated for different thresholds of Pearson correlation coefficient. Finally, we calculated representative k and P(k) values for three ranges of correlation thresholds as an average of the values at each percent level within that range. Species are represented by colors while correlation thresholds are represented by different line types. Thresholds are indicated in ranges because they represent the average vector of data from five thresholds, at 1 percent increments. **B**. Distribution of pairwise correlation coefficients of orthologs for human-mouse, human-rat and mouse-rat, for both SET 1 and SET 2. The mouse versus rat comparison exhibits a higher proportion of highly correlated orthologs than either of these species with human.

We hypothesized that orthologs from more closely related species exhibit higher expression correlation than with evolutionarily more distant species. To evaluate this, we performed a correlation analysis across all pairs of orthologs between human, mouse and rat. Figure 3B shows that there is a higher correlation between the tissues of mouse and rat than between human and rat or human and mouse, both for SET 1 and SET 2. The distribution of pairwise correlations between species across tissue types revealed a majority of positively correlated, but also some negatively correlated orthologs.

It is generally assumed that fundamental processes that secure the survival of an organism are more highly conserved and therefore will exhibit higher similarity of expression between species than secondary processes. To evaluate this, we performed a Gene Ontology enrichment analysis to identify biological processes that are enriched in the fraction of genes that show highest or lowest correlations between species. Table 1 shows the most significant biological processes for genes with Pearson correlation coefficient above 0.7 for both SET 1 and SET 2, while Table 2 represents processes for genes that have a correlation score less than -0.2 for both SET 1 and SET 2. The results from the highly correlated fraction of genes indicate that processes related to synaptic transmission as well as to blood coagulation and hemostasis are highly enriched. These results clearly reflect the clustering observed in the PCA (Figure 1). In contrast, the genes having a weak negative correlation were enriched in various seemingly unrelated processes, some of which are composed of very few genes. These results suggest that they reflect dataset specific or random effects rather than global properties.

**Table 1:** Enriched GO biological processes of genes with Pearson correlation coefficient above 0.7 in SET 1 and SET 2

**Dataset**	**GOBPID**	**Pvalue**	**OddsRatio**	**ExpCount**	**Count**	**Size**	**Term**
SET 1	GO:0007599	0.00	4.38	5.52	17	51	hemostasis, blood coagulation
SET 1	GO:0051258	0.00	6.99	2.16	9	20	protein polymerization
SET 1	GO:0048489	0.00	9.90	1.41	7	13	synaptic vesicle transport
SET 2	GO:0019226	0.00	32.77		81	275	transmission of nerve impulse
SET 2	GO:0007268	0.00	28.12		71	236	synaptic transmission

**Table 2:** Enriched GO biological processes of genes with Pearson correlation coefficient smaller than -0.2 in SET 1 and SET 2

**Dataset**	**GOBPID**	**Pvalue**	**OddsRatio**	**ExpCount**	**Count**	**Size**	**Term**
SET 1	GO:0008089	0.01	403.40	0.01	1	2	anterograde axon cargo transport
SET 1	GO:0001867	0.01	201.60	0.01	1	3	complement activation, lectinpathway
SET 1	GO:0051234	0.01	10.62	1.93	5	651	establishment of localization
SET 2	GO:0010579	0.00	0.28		3	22	positive regulation of adenylatecyclase activity by GPCR
SET 2	GO:0009218	0.00	0.10		2	8	pyrimidine ribonucleotide metabolic process

We extended our correlation analysis to study the global topology of tissue expression correlation data by comparing the degree distributions for each species. For this, we modeled our expression network as an undirected graph, where a node represents a gene and an edge is drawn between two genes if their expression profiles are correlated beyond a Pearson correlation coefficient threshold. For each percentage level, a degree distribution was calculated. We then calculated an average of five degree distributions for three different ranges of correlation coefficient values, namely from 0.85 to 0.89, 0.90 to 0.94 and 0.95 to 0.99. Figure 3A shows that the expression correlation networks of the tissue transcriptomes follow a power law connectivity distribution, that is, *n*(*k*) ∼ *k*^-*γ*^. In this study, *γ* ranged from 2.9 to 3.1 which is in the typical range for a scale free network topology [26]. Tissue correlation networks therefore are composed of hubs, where different sizes of gene sets are highly correlated in comparison to a random network. It is interesting to observe that the scale free properties prevail at higher degrees for the lower correlation ranges than for the highest correlation range. This is most likely an artifact due to the smaller number of genes remaining in the network after filtering for higher correlations. In fact, the scale free properties for SET 2, which comprises four times more orthologs than SET 1, were present at higher degrees for all three organisms.

### Integrating expression and sequence data

The typical way of inferring genes with conserved function across species is to identify orthologous clusters-sets of genes that evolved from a single common gene in the last common ancestor of the species in question [27]. While sequence conservation has proven its advantage in determining orthologous relationships, this type of analysis does not include-let alone model-the associated regulatory machinery. And indeed, orthology alone is no guaranty of function conservation: many cases are known where orthologs have diverged functionally [28]. By constrast, gene expression analysis measures the dynamic, condition-specific response of complex biological systems. Furthermore, even when the ancestral function has been retained among orthologs, because of lineage-specific duplications, such clusters can contain more than one gene per organism. Indeed, approximately 2% of the orthologous clusters from human, mouse and rat are composed of such m:n:p orthologous clusters (with m, n, p all > 1). While functional redundancy of homologs within a species exists (e.g. due to gene dosage requirements), it is generally believed that most duplicated genes carry out different functions [29-32], though the difference has recently been shown to be relatively modest [33]. We hypothesized that a combination of sequence similarity and gene expression correlation might yield the most likely correct mappings of homologous genes that carry out the same function.

**Figure 4:**
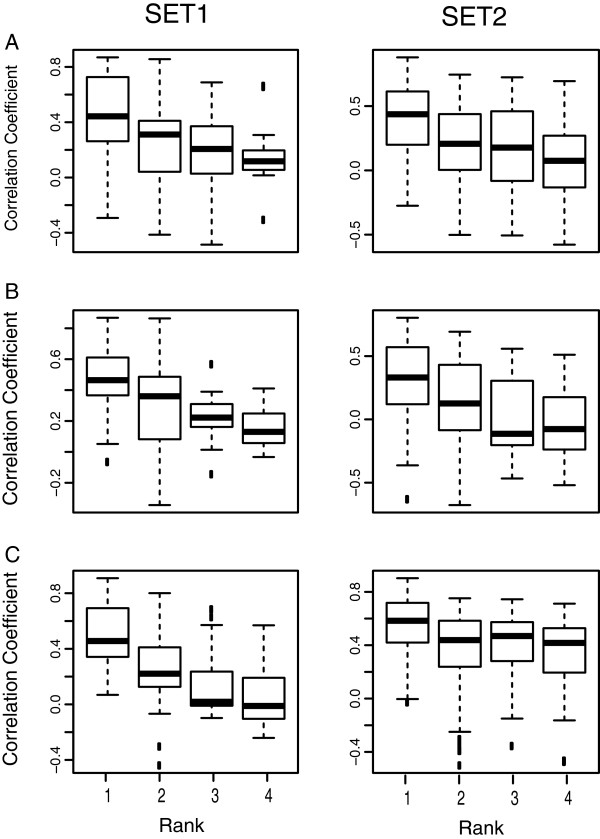
**Expression similarity in homolog clusters from human, mouse and rat. A)** Human-Mouse, **B)** Human-Rat and **C)** Mouse-Rat. For each m:n homolog cluster between two species, the correlation coefficient between each pair of genes was calculated, and the correlation coefficients were ranked from highest to lowest. Each boxplot represents all pairwise correlation coefficients having a given rank, as obtained from all homolog clusters.

For this type of analysis, we would ideally need a combination of spatial expression and transcriptional response data, since gene function is generally associated with both factors. At the level of anatomical parts, a complete overlap over a large set of tissues can be compiled, as reported here. As shown in Figures 1 and 2, tissue type signatures are highly representative of the biological processes taking place within them and contain the major source of variance between samples. Regarding transcriptional response data, a diverse but cross-species consistent dataset of responses would be required. To ensure comparability between species, these perturbations need to be carried out on the same tissue types under identical experimental conditions. Unfortunately, despite the very large number of experiments available in public repositories, the overlap of comparable perturbations between human, mouse and rat is very sparse. We therefore exploited the present dataset on tissue types to compare the pairwise correlation of expression of genes from homolog clusters. We assume that, although spatial co-location of transcripts is a partial measure of transcriptional co-regulation, it will help identifying functional orthologs from within a cluster of sequence homologs. We selected clusters having maximum four orthologous genes per species and ranked the pairwise correlations from highest (rank 1) to lowest (rank 10) for all clusters considered in this study. A graph of correlation coefficient vs rank is plotted for Human-Mouse (Figure 4A), Human-Rat (Figure 4B) and Mouse-Rat (Figure 4C) clusters.

**Figure 5:**
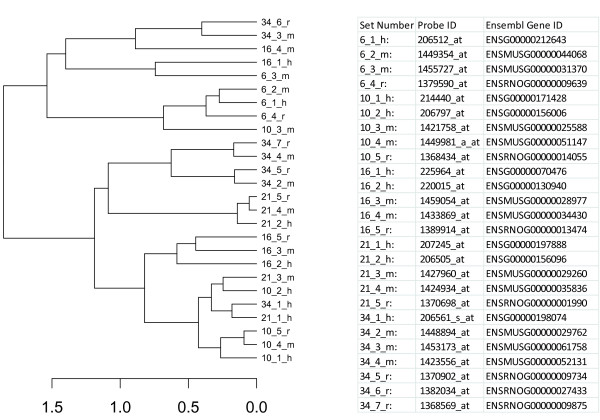
**Dendrogram representing an example where expression profiling facilitates determination of functionally related genes.** Across the tissue profiles, the gene homologs 21_2_h, 21_4_m, 21_5_r clustered together while 21_1_h as well as 21_3_m were more distant. On the sequence level alone, 21_1_h and 21_3_m had higher similarity to the rat homolog 21_5_r than the other human and mouse homologs. This example shows how combining gene expression profiles with sequence similarity helps in assigning correct mappings for functionally related genes.

The results show that the correlation coefficient between pairs of gene homologs diminishes significantly with an increase in rank, with usually the largest decrease from rank 1 to rank 2. This suggests that, for most clusters, a best pair exists that has significantly higher conservation than with any of the remaining potential functional orthologs. Based on spatial expression data alone, the results suggest that gene expression can improve the mapping of functionally related homologs (either orthologs or paralogs). For most of the cases, it was observed that gene pairs having highest sequence similarity also had high gene expression correlation, but there were several exceptions. To illustrate this, a dendrogram of expression profiles was generated for a few sets of genes, which had m:n:p relationships between organisms. In Figure 5, the homologs of the five UDP glucuronosyltransferase genes, 21_2_h, 21_4_m, 21_5_r clustered together, while 21_1_h as well as 21_3_m appeared in two distinct clusters. On the sequence level, gene 21_1_h and 21_3_m had higher similarity to the rat ortholog 21_5_r. This suggests that the UDP glucuronosyltransferase genes belonging to human and mouse have undergone a lineage-specific duplication and can be classified as paralogs. Furthermore, it appears that the less conserved sequence maintained the regulatory pattern of its functional orthologs in human and mouse, while the other is most likely involved in other processes or in the same process but under different conditions. Several previous studies have shown that duplicated genes exhibit divergent expression patterns [30-32,34]. Thus, we further investigated sets of paralogous genes that have a Pearson correlation coefficient below 0.52, a cut off used in a study by Blanc and Wolfe [34], in which they found differential gene expression for duplicated genes. The UDP glucuronosyltransferase genes belonging to human and mouse had correlation coefficient values of 0.14 and 0.50, respectively. A similar analysis on SET 1 yielded consistent results (results not shown). We conclude that by combining tissue type expression profiles with sequence similarity, we can infer improved mappings for functionally related genes as compared to using sequence information alone. Since perturbational data contains orthogonal information about gene function, the creation of such datasets which are fully comparable between human, mouse and rat would be highly desirable to further improve the inference of functional ortholog pairs.

## Conclusions

Due to the importance of both mouse and rat in biomedical research, it is imperative to study the similarities and differences relative to human, both at the level of biological processes and responses to perturbations. In this study, we focus on the first aspect. We compared a highly robust set of tissue expression profiles between orthologous genes of human, mouse and rat, obtained from the well annotated and quality controlled database Genevestigator. We used two independent data sets based on 2127 and 8954 gene clusters to substantiate our results and found that there is a strong conservation of tissue expression profiles across species for both datasets. In general, earlier studies overestimated variances between species [35], while more recent studies using larger and more standardized datasets showed a much higher level of conservation [6]. Although several previous studies compared tissue-specific gene expression profiles, none of them compared human, mouse and rat tissues systematically. In fact, most of the data used in earlier comparative studies were limited either to a relatively small number of tissue types (e.g. [5,7,16]), or to a larger but only partly overlapping set of tissues between human and mouse [6]. Nevertheless, our results are in conjunction with some of the earlier studies [6,16] and can be attributed to the conservation of functionally fundamental biological processes. In fact, during organism development, cells differentiate to reach highly complex but robust and distinct biological and functional end-states. The functional stability and homogeneity of these states is crucial for the function of larger organs and for the overall survival of the organism. Therefore, it is not surprising that these mechanisms are highly conserved between mammalian species. It was interesting to observe a stronger similarity between biologically related tissues across species than between unrelated tissues within species.

Several studies have tried to minimize platform and sample variation in order to perform cross-species comparisons (e.g. [8]). Although our analysis was performed on a dataset compiled from experiments collected from various laboratories and performed under different conditions, we observe a high conservation of tissue-specific expression. We show that variances originating from the nature of the tissue type are significantly higher than the variances arising from perturbations applied to these tissues. This has wide reaching implications on the design and sampling of biological experiments. In fact, small variations in the composition of different cell types within samples may massively bias the population of genes that appear to be responding to an external perturbation such as the challenging of cells with a chemical compound. This means that microarray or RNA-seq perturbation experiments carried out on biological samples composed of various cell types require a higher number of biological replicates than experiments carried out on single cell types such as cell culture. It also implicates that the sampling of tissue material for transcriptomic analysis should minimize the number of functionally different cell types per sample. Recent developments in single cell analytics have raised hopes of being able to eliminate such biases. However, the level of variation between neighbor cells of the same type is still a matter of debate. It seems most likely that for the measurement of cellular response to perturbations, working with a population of hundreds of identical cell types under strictly controlled conditions will yield more representative results than individual cells and require less biological replicates.

Of particular relevance are the findings about the transcriptome shift occasioned by bringing cells into culture. The use of cell cultures and cell lines to describe biological systems using RNA-seq or microarrays must be evaluated very carefully. Practically, lists of genes that are strongly biased between individual cell cultures and their tissue of origin would need to be identified, and the analysis of cell culture transcriptome data could then be significantly improved by excluding them from such analyses. It is likely that each tissue type will have a significant proportion of genes biased specifically in the corresponding cell culture, while a common set of genes, as found here, will be present for most cell types.

On the level of gene network architecture, our study showed that the tissue expression correlation networks from human, mouse and rat share common topological properties, such as scale-free connectivity distribution. Global properties like these reflect the principles underlying robustness and dynamics of these networks.

Our analysis on orthologous gene clusters having multiple orthologs revealed that gene expression profiles can improve the mapping of genes, which are functionally related. By virtue of studying tissue-specific gene expression, core sets of genes can be identified for the purpose of developing animal models of human diseases by producing transgenic rodents with tissue-specific inducible gene expression or tissue-specific gene deletions.

Finally, we report here about the spatial dimension of transcriptomes, i.e. the dimension of fundamental biological processes resulting from cell differentiation and organ development. An essential and still missing aspect in the comparative study of human, mouse and rat transcriptomes is the comparison of their response to perturbations. The generation and availability of consistent datasets from human and rodents representing a variety of perturbations carried out on the same tissue or cell types under identical conditions would be highly valuable to further our understanding and use of mouse and rat models as predictors for applications in biomedical research.

## Methods

### Orthologous gene cluster

Orthologous gene clusters of human, mouse and rat were generated from the OMA database [36]. Only clusters that have OMA IDs for all three species were considered and Ensembl gene IDs for each of the corresponding OMA IDs were retrieved from the OMA server. The mapping of Ensembl IDs to Affymetrix probe set IDs was retrieved from mapping files provided by Affymetrix ( http://www.affymetrix.com/estore/).

### Preparation of expression data

SET 1 comprised the Affymetrix platforms Human Genome U133A, Murine Genome U74 Version 2 and Rat Genome U34. These platforms contain 22283, 12654 and 3227 probe sets for human, mouse and rat, respectively. SET 2 comprised the Affymetrix platforms Human Genome U133 Plus 2.0 arrays, Mouse Genome 430 2.0 and Rat Genome 230 2.0. These platforms contain 50855, 21391 and 6870 probe sets for human, mouse and rat, respectively.

All experiments from Genevestigator are manually annotated using ontologies to ensure the controlled use of sample descriptors. The data was quality controlled as well as normalized using Robust Multi-array Average (RMA, [37]) and inter-experiment scaling (see Genevestigator User Manual; http://www.genevestigator.com). Probe sets targeting multiple transcripts represented by suffix (_x_ at, _g_at, _f_at,_r_at,_b_at,_l_at and _i_at) were filtered out and only probe sets with higher specificity represented by suffix (_at and _s_at) were taken into consideration for further analysis. In cases where multiple probesets were available for a given gene, the probe set having the maximum present call percentage across the complete database was considered. Only complete gene clusters, i.e having triplets of human, mouse and rat probe sets, were considered for further analysis.

### Data analysis

Principal Component Analysis (PCA) was carried out on a matrix, which contained the orthologous genes from the gene clusters containing 1:1:1 relationships. For each organism, expression vectors across tissue types were normalized separately using the R function *norm*. Principal Component Analysis was performed on these matrices using the *prcomp* function.

Hierarchical clustering was performed with the *hclust* function provided in R statistical package based on 1-[pearson correlation] as a distance measure and complete linkage clustering.

For analyzing the homologous gene clusters having m:n:p relationships between species, the gene clusters having a maximum of four related genes in each species were considered. For all possible combinations of the homologous genes in Human-Mouse, Human-Rat and Mouse-Rat, expression matrices were created, which were normalized and the pairwise Pearson correlation coefficient was calculated. The correlation values for Human-Mouse, Human-Rat and Mouse-Rat genes present in homologous gene clusters were then ranked from highest to lowest and the results were visualized using a box plot.

Correlation networks were constructed based on pairwise correlations between all genes of a given dataset. An edge between two genes represented by nodes was defined for correlation coefficients beyond a chosen threshold, resulting in a discretized matrix with 1, if the correlation coefficient was greater than that threshold and 0 otherwise. The degree for each gene was calculated from the matrix as a sum of number of 1s present in each row. The degree distribution was calculated by using the formula P(k) = *n*_*k*_/*n*, where *n* is the number of nodes in a network and *n*_*k*_ is the number of nodes having degree k. A degree distribution was calculated for each percent of correlation threshold, and an average of these values was calculated for each of the ranges 0.85-0.89, 0.90-0.94 and 0.95-0.99 for each species. The degree distribution per range is represented as a log-log graph of average degree distribution versus degree.

Gene Ontology enrichment analysis was performed on orthologous gene clusters with a Pearson correlation coefficient above 0.7 for both SET 1 and SET 2 and below -0.2 for both datasets. GO enrichment analysis was performed using the *GOstats* package in Bioconductor, with hypergeometric distribution testing. The complete list of genes served as the universal set. A p-value of 0.001 was considered significant for genes having positive correlation, while a p-value of 0.05 was considered significant for genes having negative correlation.

The second analysis of cell cultures versus normal tissues was carried out using the GENE SEARCH Anatomy tool from Genevestigator [18]. To allow a comparison of primary cells versus normal tissues, we excluded immortalized cell lines and blood cells from the 'target’ categories of the gene search and compared against all other normal tissues. Gene ontology enrichment was performed with GOEAST [38] and the long list of results obtained was trimmed using GO Trimming [39].

## Competing interests

The authors declare that they have no competing interest.

## Authors’ contributions

AP, SSK and PZ performed data analysis. CD, VJ, OL, SB and TH contributed in data preparation and data curation. PZ and WG supervised the research project. All authors contributed to writing the manuscript.

## Supplementary Material

Additional file 1 **Gene expression values for sets of 2127 gene orthologs of human, mouse and rat across 56 tissue type categories.** Orthologous gene sets are represented by Affymetrix probe sets from the platforms HG-U133A, MG-U74Av2, and RG-U34. Click here for file

Additional file 2 **Gene expression values for sets of 8954 gene orthologs of human, mouse and rat across 56 tissue type categories.** Orthologous gene sets are represented by Affymetrix probe sets from the platforms Human133 Plus 2.0, Mouse430 2.0, and Rat230 2.0. Click here for file

Additional file 3 Gene Ontology enrichment analysis of genes specifically expressed in the cell culture category as compared to all other tissues of SET 2. Click here for file

Additional file 4 **Output from Genevestigator using the Anatomy tool from the Gene Search toolset to identify genes specifically expressed in cell culture but minimally expressed in normal tissues.** Cell lines and blood cells were excluded from this analysis. Click here for file

Additional file 5 Gene Ontology enrichment analysis of genes specifically expressed in cell culture, but minimally expressed in normal tissues, as identified using Genevestigator. Click here for file

Additional file 6 Variance of expression of all gene orthologs within different perturbations, as obtained from Genevestigator for the platforms corresponding to SET1 and SET2. Click here for file
