# Supplementary figures and images for: Global regulatory architecture of human, mouse and rat tissue transcriptomes
Source: BMC Genomics. 2013 Oct 20;14:716. doi: 10.1186/1471-2164-14-716 (PMC4008137; doi:10.1186/1471-2164-14-716)

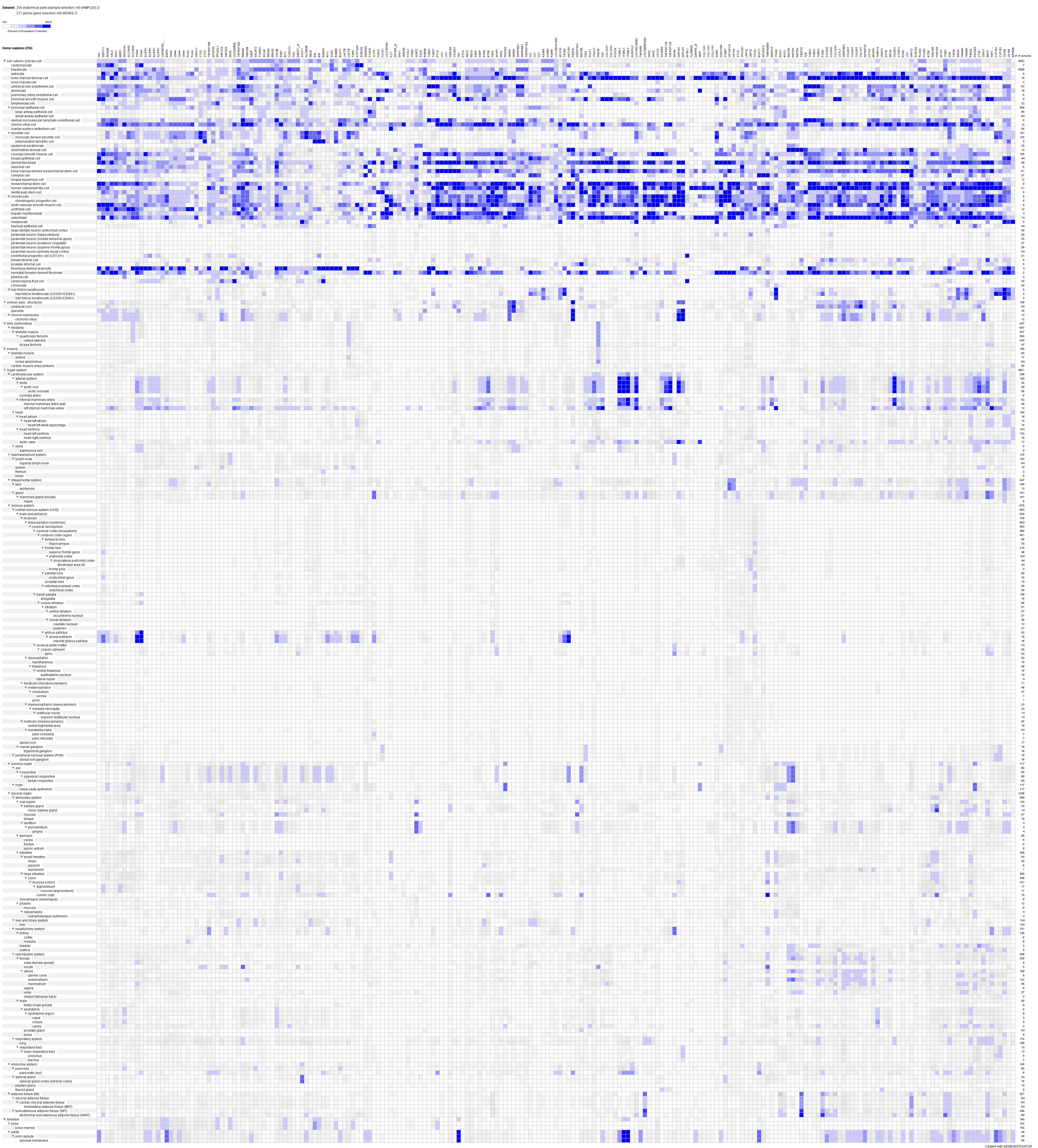

Supplement: Additional file 4 — Output from Genevestigator using the Anatomy tool from the Gene Search toolset to identify genes specifically expressed in cell culture but minimally expressed in normal tissues. Cell lines and blood cells were excluded from this analysis. [file 1471-2164-14-716-S4.png]
